# A comparative analysis of the CP and CG using 2D and 3D visualisation approaches

**DOI:** 10.1007/s12565-023-00729-3

**Published:** 2023-05-26

**Authors:** Matthew Boulton, Faith Kwa, Ali Al-Rubaie

**Affiliations:** School of Health Sciences, Swinburne University of Technology, PO Box 218, Hawthorn, VIC 3122 Australia

**Keywords:** Cribriform plate, Crista Galli, Computed tomography, 2D imaging, 3D imaging

## Abstract

Investigations on the structural variations in the cribriform plate (CP), olfactory foramina and the Crista Galli showcase the benefits of using 3D imaging on smaller structures. These techniques reveal accurate details about bone morphology and density. Comparing different techniques, this project aims to examine the correlation between the CP, olfactory foramina, and Crista Galli. Computed tomography was used to translate and apply the findings acquired from the samples in radiographic studies on CPs for potential clinical significance. The findings show that the surface area measurements were significantly larger when using 3D imaging techniques in comparison with the 2D counterpart. Using 2D imaging, the maximum surface area of the CPs was 239.54 mm^2^, however, paired 3D samples showed the maximum surface area was 355.51 mm^2^. The findings show that Crista Galli’s dimensions varied greatly, with length ranging from 15 to 26 mm, height ranging from 5 to 18 mm, and width ranging from 2 to 7 mm. The 3D imaging allowed for surface area measurements on the Crista Galli, and the surface area ranged from 130 to 390 mm^2^. When 3D imaging was used, significant correlations were found between the surface area of the CP and the length of the Crista Galli (*p* = 0.001). The findings show that measurements on the Crista Galli using 2D and 3D reconstructed radiographic imaging reflect similar ranges of dimensions to 3D imaging measurements. The findings also suggest that the Crista Galli may increase in length with the CP to support the latter and olfactory bulb during trauma which may be used by clinicians alongside 2D CT scans for optimal diagnosis.

## Introduction

The cribriform plate (CP) is a very thin bony plate that is situated in the anterior skull base and forms the roof of the nasal cavity (Gomez and Pickup 2022). The CP is lined with many foramina, called olfactory foramina, which allow the olfactory nerve fibres to travel from the nasal cavity to the olfactory bulb which rests upon the CP, and allows for the sensation of smell (Snell 2009). A large bulb-like structure called the Crista Galli separates the right and left sides of the CP, and some studies suggest that the Crista Galli may support the CP and the olfactory bulb from trauma, reducing its risk of fracture (Murray et al. 2017).

The risk of fracture is currently determined by focusing on the thinnest portion of the CP (Keast et al. 2008). These measurements are performed on the lateral lamella using various views, such as the Coronal CT scan to measure the superior-inferior length of the lateral lamella (Keros 1962), and the Axial CT scan to measure the anterior–posterior length of the lateral lamella (Yenigun et al. 2016). Lastly, the Thailand, Malaysia, and Singapore classification known as TMS classifications where distances from the orbital floor to the CP and the ethmoid roof was used (Abdullah et al. 2020). The various classifications of CP morphology are assessed using a CT scan-Coronal view; however, this may overlook many features of the CP, and increase the risk of fracture misdiagnosis.

Fractures of the CP may sever the olfactory epithelium or puncture the olfactory bulb, resulting in limited or total anosmia. Cerebrospinal Fluid (CSF) leaks may also occur due to meningeal damage or following the olfactory epithelium being severed (Kühnel and Reichert 2015), causing headaches, stroke (Shah et al. 2017) and meningitis, which can have high morbidity rates (Oh et al. 2017). Lastly, CP fractures may result in traumatic brain injuries, especially when a transnasal-penetrating injury has occurred (Gray et al. 2019; Teng et al. 2019; Yoneoka et al. 2020). These types of fractures are most commonly diagnosed using 2D CT scans (Gray et al. 2019; Douglas and White 2021; Knížek et al. 2021), however, in recent years 3D Reconstructed CT scans have been used to diagnose CP fractures (Teng et al. 2019).

As technology advances, 3D imaging techniques have increased in use amongst clinicians as it allows for precise diagnosis of fractures to be performed in the facial region (Wei et al. 2015; Teng et al. 2019, Wubulihasimu et al. (2021). Recent examples include a recent study that categorized anterior skull base fractures using virtual reality 3D hologram technology (Umana et al. 2022). In clinical case studies, 3D reconstructed CT scans have been used to diagnose CP fractures and the resulting CSF leaks have damaging consequences (Teng et al. 2019). Many studies suggest that the preciseness of 3D imaging techniques may allow for more accurate measurements to be performed on anatomical structures compared to measurements determined by 2D imaging or 2D CT scans (Wei et al. 2015; Teng et al. 2019; Belgin et al. 2021, Wubulihasimu et al. 2021).

With many of the current classification systems measuring the lateral lamella (Keros 1962; Yenigun et al. 2016) and the nasal cavity (Abdullah et al. 2020), limited attention has been given to other features of the CP, such as its surface area, olfactory foramina and bone percentages, or the Crista Galli’s dimensions. While advanced 3D imaging has been used on the CP to diagnose fractures (Teng et al. 2019), limited studies have utilised this technology to perform measurements on the CP.

This project aims to use multiple imaging techniques on human samples to investigate the variation and correlation between the CP, olfactory foramina, and Crista Galli. It also used computed tomography (CT) scans to translate and apply the findings acquired from 2 and 3D imaging into radiographic studies of CPs to determine the potential clinical significance of the measurements.

It is hypothesised that measuring the surface area of the CP by using 3D imaging will result in a larger calculated surface area than when 2D imaging is used also, the morphology of the CP will positively correlate with the dimensions of the Crista Galli. Lastly, the measurements using 3D imaging will result in similar dimensions to radiographic studies on the CP and Crista Galli.

## Materials and methods

### Experimental design

The authors hereby confirm that every effort was made to comply with all local and international ethical guidelines and laws concerning the use of human cadaveric donors in anatomical research. A total of 42 samples from human tissue donors were received with approval from the Swinburne Human Ethics Committee (Human Ethics Approval Number #2026045-9453). To include matched sample pairs to compare the 2D and 3D imaging methods, images were excluded if the 3D imaging calibration failed, or if the CP surface area was impaired preventing accurate measurements. Further analysis was then conducted using five donor CT scans of CPs which were acquired from Digital Imaging and Communications in Medicine (DICOM). This number of samples was used as they were the only CT scans available that captured the CP and Crista Galli.

The 2D images of the samples were captured from the superior view using a Canon Digital Camera DSLR EOS 1100D, with a ruler at the same level as the CP to allow for accurate calibration and scaling of length measurements. For the duration of this study, the images were stored in a secure Swinburne drive which is only accessible by authorised researchers.

These images were then uploaded to ImageJ (Wayne Rasband, NIH, Bethesda, MD, USA) to allow measurements to be performed and for images to be calibrated so those accurate length measurements can be made. Each image was then individually calibrated by measuring 10 mm on the ruler at the same level of the CP, with the number of pixels representing 10 mm calibrated into the image.

**Fig. 1:**
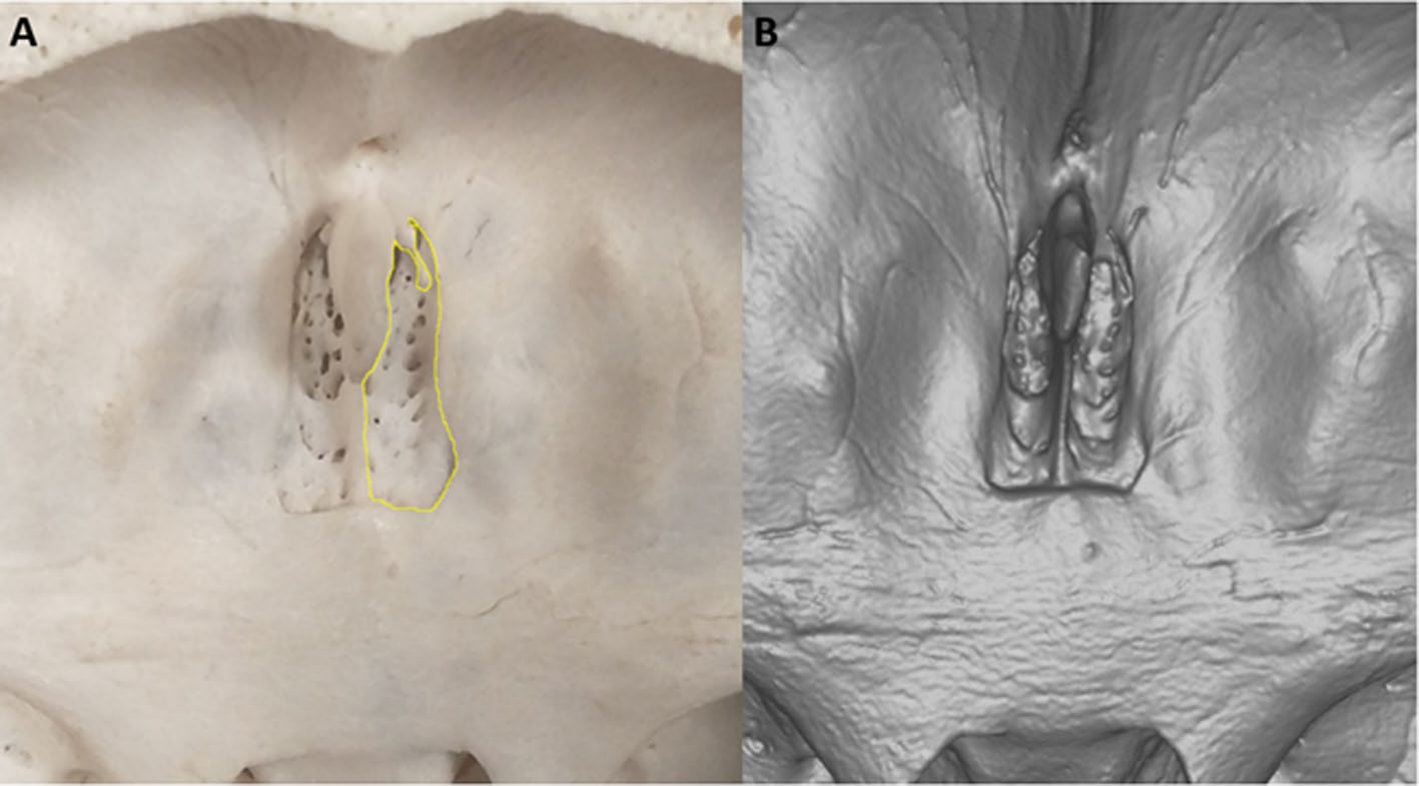
**A** Superior view image of Cribriform Plate showing right surface area traced using ImageJ (Wayne Rasband, NIH, Bethesda, MD, USA). **B** 3D Image of Cribriform Plate looking from Superior View using ArtecStudio (Luxembourg)

All measurements were performed by two blinded researchers, with the resulting measurements being averaged for the final data. Measurements were then carried out on the surface area of the CP. This was measured by tracing the borders of the CP (see Fig. 1A), and then ImageJ was used to automatically calculate the surface area of the encircled region in mm^2^. This was conducted on both the left and right sides of the CP and was summed for the total area.

The total olfactory foramina area of the CP was measured by tracing the borders of the foramina of the CP, which was then automatically calculated in mm^2^ using ImageJ. The 2D foramina percentage was then calculated by dividing the 2D foramina area by the total 2D surface area, multiplied by 100%. The remainder of the area of the CP which was not represented by foramina was then recorded as the 2D bone percentage.

Three-dimensional images for the same samples were also captured using the Artec Space Spider and were automatically calibrated to allow for accurate measurements to be performed on the scans. These images were analysed using ArtecStudio (Luxembourg) for measurements to be performed, as shown in Fig. 1B.

Measurements were made on the surface area of the left and right sides of the CP, which was then summed for the total surface area. The surface area was measured by highlighting the CP region. Using ArtecStudio (Luxembourg), the surface area of the highlighted region was automatically calculated in mm^2^.

Further measurements were conducted on the Crista Galli’s total length, width, and height in mm, alongside the total surface area in mm^2^. These measurements on the Crista Galli were performed firstly by highlighting the Crista Galli region, then excluding all structures from the 3D model except the Crista Galli. The surface area of the Crista Galli was automatically calculated, and then direct measurements were performed to measure its length, width, and height. The 3D foramina percentage was calculated by dividing the 2D foramina area by the total 3D surface area of the CP multiplied by 100%. The remainder of the area of the CP not represented by foramina was then recorded as the 3D bone percentage.

### Radiological analysis

**Fig. 2:**
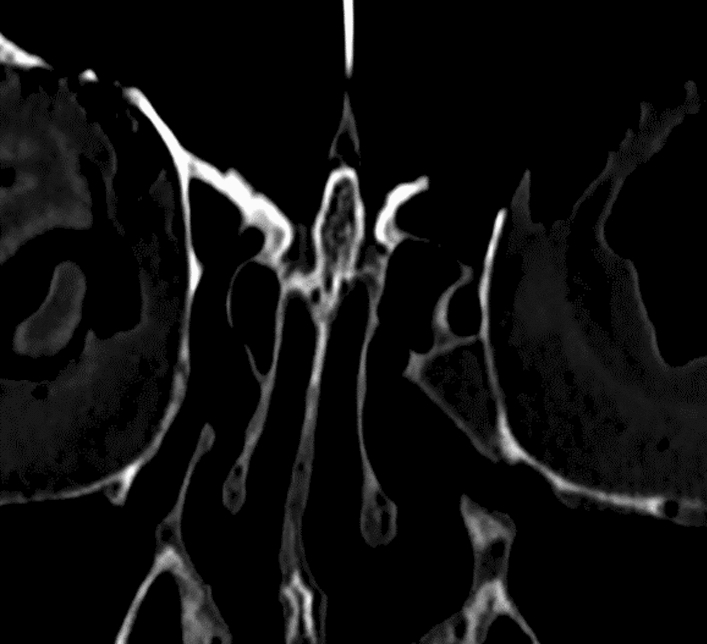
Coronal view CT scan of Crista Galli and Cribriform Plate in Bone Window mode using SECTRA (Linköping, Sweden) IDS7 Software

**Fig. 3:**
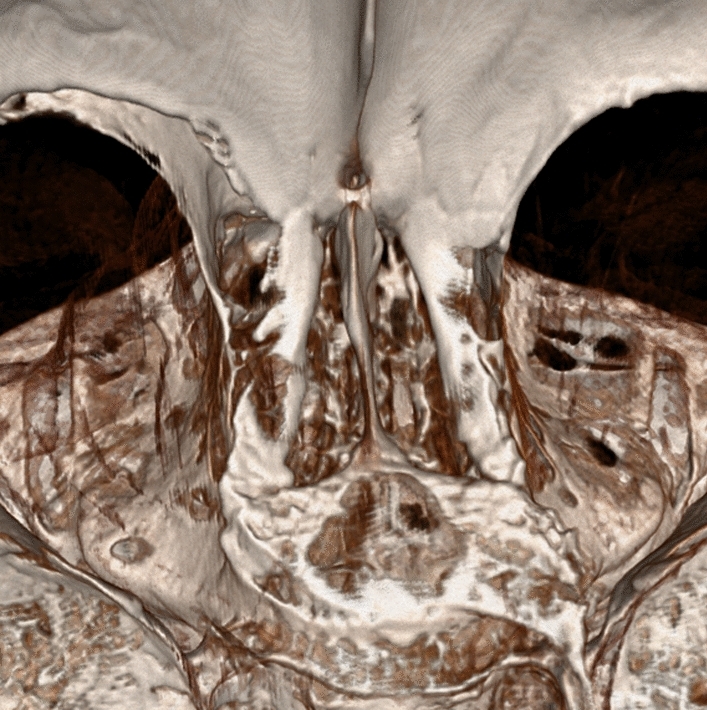
3D Reconstructed CT scan of Cribriform Plate and Crista Galli using SECTRA (Linköping, Sweden) IDS7 Software

For the radiological samples, the above-mentioned five multiplanar CT scans were used to conduct further measurements. The CT scans images were received using Digital Imaging and Communications in Medicine (DICOM) data with a slice thickness of 0.8 mm. These CT scans were measured using SECTRA (Linköping, Sweden) IDS7 software using Coronal View CT scans as shown in Fig. 2 and 3D reconstructed CT scans as shown in Fig. 3.

Measurements were conducted on the Crista Galli dimensions. The Crista Galli’s height was measured using Coronal CT scan sections as seen in Fig. 2, with five separate cuts of the Crista Galli being measured, the greatest height was recorded. Similarly, the width of the Crista Galli was measured using Coronal CT scan sections, with five separate cuts of the Crista Galli being measured, and the greatest width was recorded. The length of the Crista Galli was measured using 3D reconstructed CT scans as seen in Fig. 3.

### Statistical analysis

Statistical analysis was then conducted on the collected measurements using SPSS (ver. 28.0.1.1; SPSS Inc., Chicago, IL, USA) and GraphPad Prism 7 software (San Diego, CA, USA). To compare the 2D CP surface area to the 3D CP surface area, paired samples *t* tests were performed with *p* < 0.05 being considered a statistically significant difference. To compare the CP’s 2D bone percentage with the 3D bone percentage, paired samples t-tests were performed with *p* < 0.05 being considered a statistically significant difference. Lastly, to analyse any correlations between the Crista Galli and the CP’s surface area or olfactory foramina area, bivariate correlations were performed with *p* < 0.05 being considered a statistically significant correlation.

## Results

2D image analysis was performed on the surface area of the CP. The results showed the samples ranged between 100.11 and 239.54 mm^2^, with an average surface area of 152.60 mm^2^ (SD 33.72) as shown in Table 1. Using the same paired samples, 3D image analysis was conducted on the CP’s surface area. The results showed the samples ranged between 205.82 and 355.51 mm^2^, with an average of 288 mm^2^ (SD 46.10) as shown in Table 1.

**Table 1:** CP surface area measurements using 2D imaging and 3D imaging, foramen area measurements using 2D imaging, and the resulting bone percentages for 2D and 3D surface area (*N* = 23)

Dimension measured	Mean and SD	Minimum	Maximum
2D surface area (mm^2^)	152.60 ± 33.72	100.11	239.54
3D surface area (mm^2^)	288.00 ± 46.10	205.82	355.51
2D foramen area (mm^2^)	17.19 ± 5.00	10.23	30.92
2D bone percentage	88.41% ± 3.63%	81.62%	93.01%
3D bone percentage	93.96% ± 1.67%	90.84%	96.63%

*SD* standard deviation, *Min.* minimum value, *Max*. maximum value, *N* number of samples

**Fig. 4:**
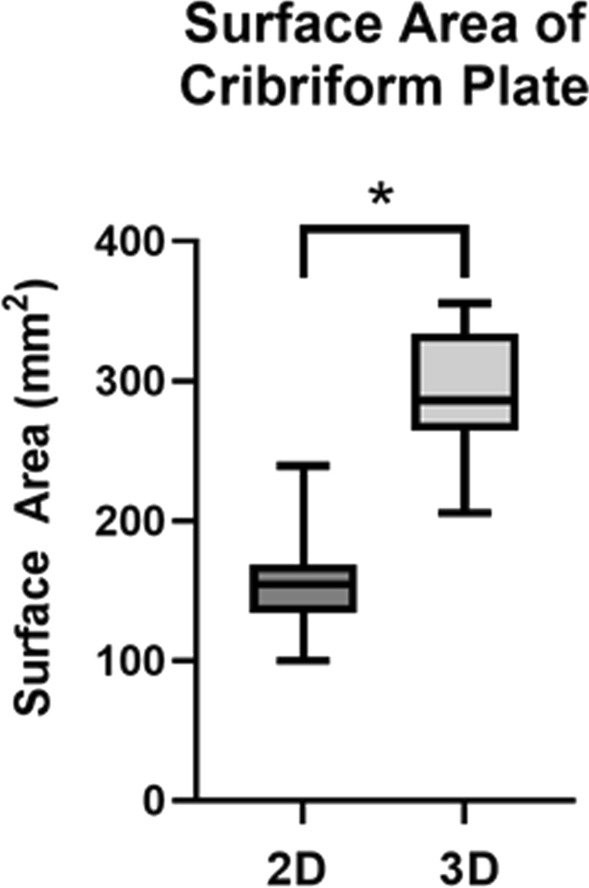
Box plot comparing 2D cribriform plate surface area with 3D cribriform plate surface area, n=23 (*p < 0.05). The data shown here are represented in Table 2

The results showed that for the 23 paired CP samples, the 2D surface area ranged from 100.11 to 239.54 mm^2^, while the 3D surface area ranged from 205.82 to 355.51 mm^2^, with the comparison being shown in Fig. 4. The difference in surface area measurements between 2D and 3D imaging was compared using a paired samples *t* test, which was determined to be statistically significant (*p* < 0.001). This test determined that the 3D imaging surface area measurements were significantly higher than the 2D imaging surface area measurements. A strong significant positive correlation was also found between the 2D and 3D surface area measurements (*r* = 0.811, *p* < 0.001).

Using the same 23 human tissue samples, 2D image analysis was performed on the foramen area of the CP. The results showed the foramen area ranged between 10.23 and 30.92 mm^2^, with an average foramen area of 17.19 mm^2^ (SD 5) as shown in Table 1.

To calculate the bone percentage of the CPs, the area of the olfactory foramina was divided by the total surface area of the CP measured using 2D imaging and 3D imaging, multiplied by 100%. This gave the percentage of the CP represented by foramen for both the 2D surface area and the 3D surface area. The remaining surface area in the CP was then presumed to be bone, resulting in a 2D bone percentage (using 2D surface area) and a 3D bone percentage (using 3D surface area). In general, the 2D bone percentages ranged between 81.62 and 93.01% bone, with an average bone percentage of 88.41% (SD 3.63%), while the 3D bone percentages ranged between 90.84 and 96.63%, with an average bone percentage of 93.96% (SD 1.67%) as shown in Table 1.

**Fig. 5:**
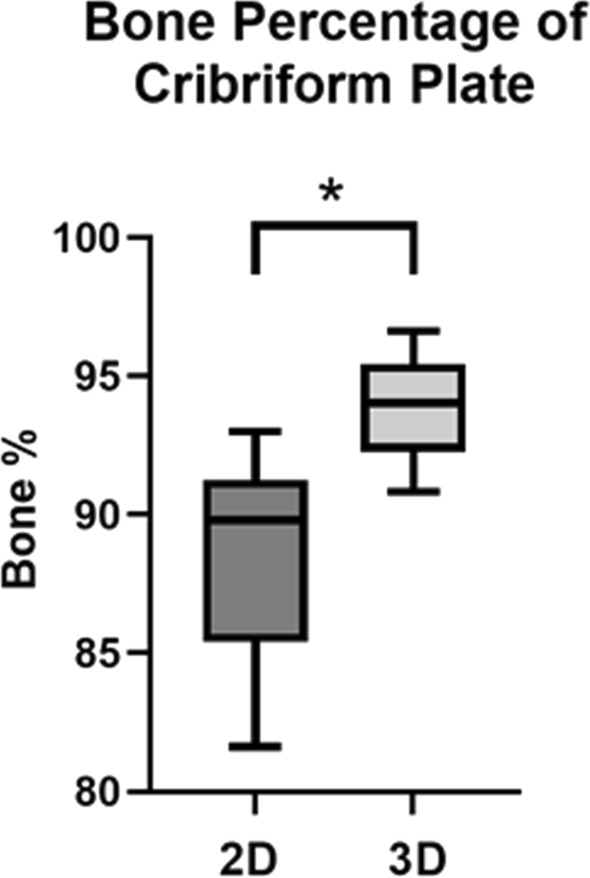
Box plot comparing 2D cribriform plate surface area with 3D cribriform plate surface area, *n* = 23 (*p < 0.05). The data shown here are represented in Table 2.

The results showed that in the 23 paired CP samples, the 2D bone percentages ranged from 81.62 to 93.01%, while the 3D bone percentages ranged from 90.84 to 96.63%, which can be seen in Fig. 5. The difference in bone percentage measurements using 2D and 3D surface area was compared using a paired samples *t* test, which was determined to be statistically significant (*p* < 0.001). This test determined that the resulting bone percentages calculated using 3D surface area were significantly higher than the bone percentages calculated using 2D surface area. A strong significant positive correlation was also found between the 2D and 3D bone percentage measurements (*r* = 0.918, *p* < 0.001).

Using a total of 35 human tissue samples, 3D imaging (ArtecStudio) was used to measure the Crista Galli’s length, height, width, and surface area. The results show that Crista Galli’s length ranged from 15.8 to 25.8 mm, with an average length of 20 mm (SD 2.45), Crista Galli’s height ranged from 5.3 to 17.7 mm, with an average height of 11 mm (SD 2.70), the Crista Galli’s width ranged from 2 to 6.6 mm, with an average width of 4.4 mm (SD 1.03) and the Crista Galli’s surface area ranged from 130 to 390 mm^2^, with an average surface area of 217 mm^2^ (SD 56.56). The measurements of Crista Galli’s dimensions using 3D imaging can be seen in Table 2.

**Table 2:** Measurements on the Crista Galli’s length, height, width and surface area measured using 3D imaging (ArtecStudio) (*N* = 35)

Crista Galli dimension	Mean and SD	Minimum	Maximum
Length (mm)	19.96 ± 2.45	15.84	25.79
Height (mm)	10.75 ± 2.70	5.34	17.68
Width (mm)	4.41 ± 1.03	2.04	6.61
Surface area (mm^2^)	217.36 ± 56.56	135.62	384.85

*SD* standard deviation, *Min*. minimum value, *Max*. maximum value, *N* number of samples

Using the 23 paired CP samples, bivariate correlations were run between the Crista Galli’s dimensions measured using 3D imaging and the CP’s total surface area measurements, to determine whether the Crista Galli’s dimensions increased in size alongside the CP’s surface area. There were no significant correlations found between the surface area of the Crista Galli and the surface area of the CP when measured using 2D (*p* = 0.968) or 3D imaging (*p* = 0.498). Furthermore, there were also no significant correlations between the length of the Crista Galli and the CP’s total surface area using 2D (*p* = 0.119) or 3D imaging (*p* = 0.067).

When the surface area of the CP was divided between the left and right sides, a significant moderate positive correlation was found between the surface area of the left side of the CP and the length of the Crista Galli, when the surface area was measured using both 2D (*r* = 0.549, *p* = 0.007) and 3D imaging (*r* = 0.634, *p* = 0.001).

There were significant negative correlations between Crista Galli’s width and the surface area of the CP. This correlation was significant when the surface area was measured using 2D (*r* = − 0.532, *p* = 0.009) and 3D imaging (*r* = − 0.429, *p* = 0.041).

There were no significant correlations found between the height of the Crista Galli and the CP surface area when measured using 2D (*p* = 0.588) or 3D imaging (*p* = 0.228).

### CT scan

**Fig. 6:**
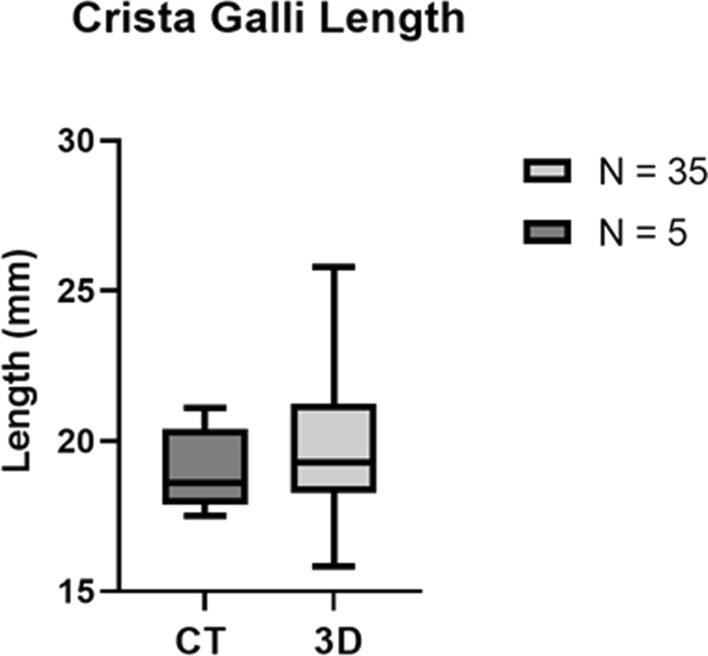
Box plot comparing the distributions of the Crista Galli’s length using ArtecStudio 3D imaging (n=35) and 3D reconstructed CT scans (*n* = 5). The data shown here is represented in Tables 2 and 3

**Fig. 7:**
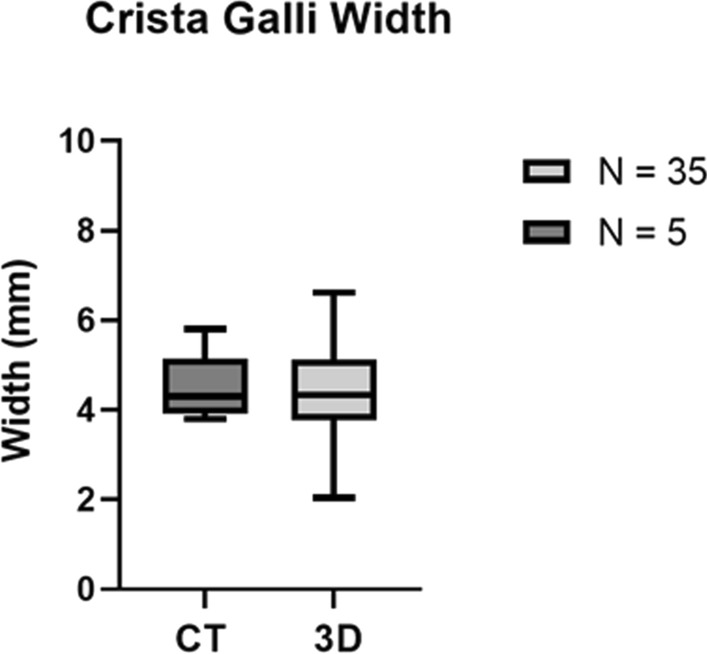
Figure 7. Box plot comparing distributions of the Crista Galli’s width when measured using ArtecStudio 3D imaging technology (*n* = 35) and Coronal view CT scan technology (*n* = 5). The data shown here are represented in Tables 2 and 3

**Fig. 8:**
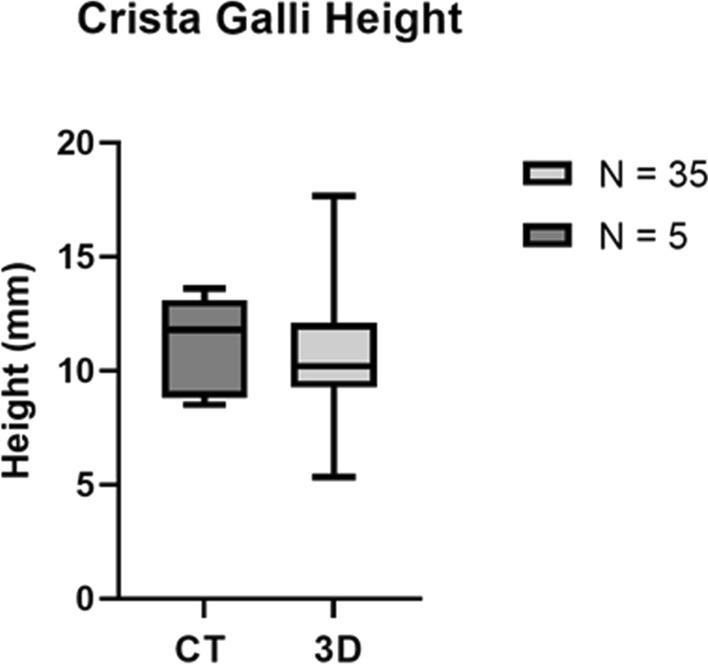
Box plot comparing distributions of Crista Galli’s height when measured using ArtecStudio 3D imaging technology (n=35) and Coronal view CT scan technology (*n* = 5). The data shown here are represented in Tables 2 and 3

Five CT scans from human donors were used, with measurements being performed on the Crista Galli’s length, width, and height in Hounsfield units in the bone window. The results show that Crista Galli’s length ranged between 17.5 and 21.1 mm, with an average length of 19.04 mm (SD 1.40). The Crista Galli’s width ranged between 3.8 and 5.8 mm, with an average width of 4.48 mm (SD 0.79). Lastly, Crista Galli’s height ranged between 8.5 and 13.6 mm, with an average height of 11.12 mm (SD 2.22). The measurements of Crista Galli’s dimensions measured using CT scans can be seen in Table 3. The range of the Crista Galli’s dimensions which were measured using CT scans (SECTRA ISD7) was consistent with the range of Crista Galli’s dimensions measured using 3D imaging (ArtecStudio). This was true for Crista Galli’s length shown in Fig. 6, Crista Galli’s width shown in Fig. 7, and Crista Galli’s height shown in Fig. 8.

**Table 3:** Crista Galli’s length, height, and width were measured using CT scan technology (*N* = 5)

Crista Galli dimension	Mean and SD	Minimum	Maximum
Length (mm)	19.04 ± 1.40	17.50	21.10
Width (mm)	4.48 ± 0.79	3.80	5.80
Height (mm)	11.12 ± 2.22	8.50	13.60

*SD* standard deviation, *Min*. minimum value, *Max*. maximum value, *N* number of samples

## Discussion

Recent medical literature shows a significant increase in the use of 3D imaging as a diagnostic tool in many clinical cases, including facial fractures (de Carvalho et al. 2021, Wickwire et al. 2022), naso-orbital-ethmoid fractures (Onisor 2022), cervical fractures (Ren et al. 2021) and orbital blowout fractures (Jansen et al. 2018). Researchers have also used this technology to conduct virtual surgery on the scaphoid bone (Faudot et al. 2021) while other studies have used it to scan the peri-orbital region for a very high clinical accuracy (Hollander et al. 2021).

This project used human tissue samples and advanced imaging technology in the form of two-dimensional (2D) and three-dimensional (3D) imaging to investigate the variation and correlations between the CP and the olfactory foramina, alongside examining the dimensions of the Crista Galli in correlation with the CP. This study also used CT scan technology to translate and apply the findings from the above aims into radiographic studies of CPs to determine the potential clinical significance of the measurements.

To establish a complete set of measurements, the following techniques were used to measure and correlate the dimensions of the CP, olfactory foramina, and Crista Galli. The 2D imaging technique was chosen as it is used in much of the current literature when direct anatomical measurements are being performed on dry skull samples (Coelho et al. 2018; Ganjaei et al. 2018). This imaging technique used calibrated digital images of CPs. This was compared with the 3D imaging technique, an advanced imaging technique that was chosen as a comparative measure to determine the potential benefits this technology may have on diagnosis and measurements on smaller structures such as the CP (Teng et al. 2019). This technique used an advanced 3D scanner (ArtecStudio) to measure the dimensions of the CP and the Crista Galli. The CT scan data was measured using Coronal CT scans, as this is the most common method of radiographic measurement on the CP (Keros 1962; Skorek et al. 2017; Inal et al. 2018, Abdullah et al. 2020) and the Crista Galli (Şahan et al. 2019; Keşkek and Aytuğar 2021). Lastly, the CT scan data were then measured using 3D reconstructed CT scans to measure the greatest length of the Crista Galli. This was chosen to accurately measure the entire length of the Crista Galli, as opposed to the axial view used in other studies (Akiyama and Kondo 2020) which may not accurately represent the full length of the Crista Galli. The morphology of the CP is concave in shape to house the olfactory bulb and the numerous olfactory foramina that pass through the CP, from the nasal cavity to the olfactory bulb (Snell 2009). This curvature in the morphology cannot be detected using 2D imaging, however, the 3D scanners used in this study enabled a more specific and accurate measurement method that traces the curvature of these samples.

This study measured the CP’s total surface area using superior view 2D images and 3D imaging technology, to compare the differences in surface area measurements of these two imaging techniques. The measurements performed on the surface area of the CP using 3D imaging were significantly higher than the measurements performed using 2D imaging. This difference in measurements supports the hypothesis that the surface area of the CP would be greater when measured using 3D imaging compared to 2D imaging. The resulting greater surface area of the CP measured by 3D imaging may indicate that prior studies which have investigated the surface area and foramina area of the CP using 2D images (Ganjaei et al. 2018) did not accurately depict the surface area of the CP. This is due to the 2D imaging method not accurately measuring the additional surface area that the curvature of the CP may provide, thus resulting in a lower overall surface area, and a lower calculated bone percentage than would otherwise be found.

Using 2D imaging to measure total surface area, the proportion of the CP which was represented by olfactory foramina ranged from 7 to 19% of the CP, resulting in roughly 81–93% of the CP area consisting of bone. This is a similar range to previous research which suggests found foramen areas ranging from 9 to 26%, resulting in roughly 74–91% of the CP consisting of bone (Ganjaei et al. 2018). However, due to the increased surface area measurements from using 3D imaging which accounts for the curvature of the CP, the proportion of foramina to the bone area of the CP ranged from 3 to 10%, which resulted in 90–97% of the CP consisting of bone. This value is considerably less than previous research using anatomical specimens (Ganjaei et al. 2018), which may demonstrate that additional measurements need to be taken to accurately determine the foramina to bone ratio when measuring using 2D imaging.

The measurements of the bone percentage of the CP using the 3D surface area resulted in significantly higher bone percentages than when the bone percentage was calculated using the 2D surface area. This suggests that the 2D imaging technique may not accurately represent the bone percentage of the CP, as it does not account for the additional surface area from the curvature of the CP which is obtained when 3D imaging is used.

The additional surface area measured using 3D imaging techniques resulted in the majority of 3D imaging samples recording 60–140% additional surface area when using 3D imaging compared to their 2D imaging counterparts. This shows a large variation in the additional surface area resulting from the curvature of the CP, and this curvature has not been well studied in the current literature due to limited access to 3D imaging technology. This difference in the surface area suggests that the 2D imaging technique is insufficient to accurately measure the full surface area of the CP, and results in much lower bone percentages than would otherwise be reported. This finding also impacts our current knowledge of the CP’s olfactory foramina, as there may be vastly fewer foramina reported by 2D imaging methods.

Prior research by Ganjaei et al. (2018) compared the recorded bone percentage of the CP using 2D imaging on anatomical samples with the bone percentage measured using CT scans, with the recorded bone percentage using a CT scan ranging from 92 to 98% with an average of 95.3% bone. These results are quite similar to the bone percentages calculated in this study when accounting for the curvature of the CP by measuring surface area using 3D imaging, which ranged from 90 to 97% with an average of 94% bone. Initially, their study concluded that the bone percentages recorded using CT scans, which averaged 95.3% were inaccurate due to the differences in bone calculations of roughly 11% when compared with the paired 2D bone percentage which averaged 84.5% (Ganjaei et al. 2018). However, due to the similarities with the range found in this study, the results may suggest that measuring bone percentage using CT scans may include the curvature of the CP in its measurements. This suggests that CT scans may provide a more accurate depiction of the bone percentages than when measurements are conducted using 2D imaging.

Researchers who may face difficulties in accessing 3D imaging ArtecStudios may be able to use CT scans to measure the bone percentage of the CP. This data could be compared to 2D imaging findings to determine the additional surface area for which the curvature is included.

For clinical cases, 3D reconstructed CT scans would be beneficial to use, however, this view would require a high level of radiographic exposure to study the CP, Crista Galli, and olfactory foramina in detail. When a facial fracture has occurred, due to the susceptibility of the CP to fracture (Kühnel and Reichert 2015), clinicians could conduct additional checks to determine whether an individual’s CP is at a higher risk of fracture. This could be useful to prevent potential spontaneous CSF leaks (Murray et al. 2017) and the infections which may occur as a result (Oh et al. 2017; Stopa et al. 2022).

The length of the Crista Galli measured using 3D imaging ranged from 15.8 to 25.8 mm, these results are similar to previous research showing large variability of Crista Galli length (Akiyama and Kondo 2020). However, the minimum length of this study was considerably less than that of previous reports (33). This difference may be due to the different measuring methods used, as the studies which measure the length of the Crista Galli commonly use Axial CT scans (Akiyama and Kondo 2020), which may be unable to measure the entire length as accurately as 3D imaging. Due to the variations in the height of the Crista Galli, one section of the axial view may only be able to measure a portion of the length, resulting in a much smaller length measurement than would otherwise be reported for that sample if 3D imaging was used. This shows the benefits of using 3D imaging for measurement, as this enabled much more accurate length measurements to be made.

The height of the Crista Galli measured using 3D imaging ranged from 5.3 to 17.7 mm, similarly supporting previous research indicating a large range of height measurements from the Crista Galli (Akiyama and Kondo 2020). However, the recorded maximum range of 17.7 mm was less than the maximum of 26 mm found by Akiyama and Kondo (Akiyama and Kondo 2020). This difference is likely due to sampling size, as the previous study by Akiyama and Kondo (Akiyama and Kondo 2020) had a much larger sample size of 300, allowing for a larger range of Crista Galli’s height to be recorded.

The Crista Galli’s width measured using 3D imaging ranged from 2 to 6.6 mm, which is quite similar to previous research which has found this width to range from 0 to 4 mm (Coelho et al. 2018), 2–10 mm (Akiyama and Kondo 2020) and 2.5–7 mm (Şahan et al. 2019). The width of the Crista Galli may impact the risk of trauma-related fracture, as it has been suggested to provide support to the CP (Murray et al. 2017). From this, it can be postulated that an increased width of the Crista Galli may result in a decreased risk of trauma-related fracture, due to the buttressing effect that it may have (Murray et al. 2017). The increased width of the Crista Galli would also likely protect the olfactory bulbs which rest upon the anterior portion of the CP from trauma.

The surface area of the Crista Galli using 3D imaging ranged from 135.6 to 384.9 mm^2^. An increase in the surface area of the Crista Galli and the resulting volume may help insulate the CP from fractures that may occur and limit the resulting damage which may be inflicted on the olfactory bulb. Due to the wide range of Crista Galli’s surface area, this implies vastly differing levels of insulation to fracture that the Crista Galli may be providing between individuals. This variation may be able to be classified to determine the risk of fracture, with a larger surface area or volume of the Crista Galli resulting in a decreased risk of fracture and a lowered risk of trauma to the olfactory bulb.

This study found a strong significant positive correlation between the surface area of the CP when measured using 2D imaging and 3D imaging (*r* = 0.811, *p* < 0.001), alongside a strong significant positive correlation between the 2D and 3D bone percentages (*r* = 0.918, *p* < 0.001). These correlations show the similarities between the measurements of 2D and 3D imaging, which may provide support for the accuracy of the 3D imaging method, while also displaying significantly higher surface area measurements and bone percentage calculations.

There was no significant correlation between the total surface area of the CP and Crista Galli length. When data were used to contrast the difference between the left and right sides, this resulted in the left surface area of the CP having a moderate positive correlation with the length of the Crista Galli for both 2D imaging (*r* = 0.549, *p* = 0.007) and 3D imaging (*r* = 0.634, *p* = 0.001). This correlation suggests that as the Crista Galli increases in length, so does the surface area of the left side of the CP. These results may suggest that as the surface area of the CP increases, so does the Crista Galli’s length, to support the CP (Murray et al. 2017) and protect the olfactory bulb from trauma. The ability to measure accurately and efficiently the length of the Crista Galli to determine the size of the CP may assist clinicians when conducting pre-operative assessments. This could allow them to determine the size of the CP, to avoid surgical complications which may occur if surgery is being conducted close to the CP, such as during endoscopic sinus surgery (Abdullah et al. 2020) and ethmoidectomy (Goanţǎ et al. 2017).

A moderately significant negative correlation was also found between the total surface area of the CP and Crista Galli’s width. This correlation was found when the surface area was measured with both 2D imaging (*r* = − 0.532, *p* = 0.003) and 3D imaging (*r* = − 0.429, *p* = 0.041). This likely resulted from the Crista Galli blocking the anterior portion of the CP’s surface area (Kalmey et al. 1998; Patron et al. 2015; Ganjaei et al. 2018), lowering the total measured surface area of the CP. The Crista Galli’s width could be explored in more detail in a dissection study, whereby the dimensions of the Crista Galli could be recorded, then subsequently removed, and the CP’s surface area then measured to more accurately assess whether the Crista Galli’s width correlates with the total surface area of the CP.

There were no significant correlations between the CP’s total surface area and the Crista Galli’s height, or surface area. The length, height, and width of the Crista Galli also did not significantly correlate with each other, suggesting that these dimensions develop separately and do not impact each other.

The Crista Galli’s length measured using a 3D Reconstructed CT scan ranged from 17.5 to 21.1 mm, which is consistent with the range of length measurements recorded using 3D Imaging of 15.8–25.8 mm. This suggests a high accuracy of measurements for both 3D CT scans alongside the 3D imaging used in this study. The length measurements recorded using the 3D Reconstructed CT scan were similarly much greater than the minimum recorded using Axial CT sections by Akiyama and Kondo (Akiyama and Kondo 2020). This again supports the use of 3D imaging, with 3D Reconstructed CT scans giving a similar range of length measurements to the 3D imaging, and accurately representing the length of the Crista Galli compared to the Axial view.

Crista Galli’s height measured using Coronal CT scans ranged from 8.5 to 13.6 mm, which is consistent with the range of height measurements recorded using 3D imaging of 5.3–17.7 mm. This was also consistent with the range recorded in previous studies of 5–21 mm (Şahan et al. 2019; Akiyama and Kondo 2020), which further supports the accuracy of the 3D imaging technique and the use of Coronal CT scans to accurately measure the height of the Crista Galli.

The Crista Galli’s width measured using Coronal CT scans ranged from 3.8 to 5.8 mm, which is consistent with the range of width measurements recorded using 3D imaging of 2–6.6 mm. This was also consistent with the range recorded in previous studies of 2–10 mm (Akiyama and Kondo 2020), 2.5–7 mm (Şahan et al. 2019), and 0–4 mm (Coelho et al. 2018). While studies have measured the width of the Crista Galli using a variety of methods such as Coronal CT scans (Şahan et al. 2019), Axial CT scans (Akiyama and Kondo 2020), and 2D imaging (Coelho et al. 2018), the similarities between these results suggest that the width of the Crista Galli can be accurately determined through a variety of imaging techniques.

## Conclusion

The results of this study support the use of 3D imaging techniques, alongside the 3D reconstructed CT scan as an accurate method for measurement, which allows for comprehensive analysis of complex surfaces such as the CP morphology and variations, olfactory foramina, and Crista Galli. This study shows the potential correlations between the Crista Galli and the CP, with a positive correlation between the length of the Crista Galli and the surface area of the left side of the CP. This correlation should be further investigated, as this may suggest that the Crista Galli increases in length alongside the CP to support the bone and olfactory bulb. The similarities between the results using 3D imaging, CT scans, and 3D reconstructed CT scans also support the use of these imaging techniques as a precise measurement tool for researchers, which could be used to assess future risk of trauma in further studies. For clinicians, 3D reconstructed CT scans could be used alongside 2D CT scan technology to perform accurate diagnoses, as this may allow for a more precise and holistic assessment to be performed on patients when head injuries have occurred.

The demographic data and patient information of the CP samples could not be accessed, and detailed data analysis (e.g., how features such as age, sex and race may impact the morphology of the CP and the Crista Galli) on these features was unable to be conducted. However, studies measuring Crista Galli’s dimensions with regard to age would be of interest for future studies due to the implications on the risk of fracture. Future research may investigate these findings with a larger samples size to confirm the correlations which may occur between the Crista Galli and Cribriform Plate.

The implementation of simulated fracture technology to directly investigate the risk of trauma-related fracture of the CP and its structures could benefit the literature, with this technology already being used on the femur, humerus, fibula, and ulna (Ota et al. 1999; Parra-Cabrera et al. 2022). Researchers could also measure the bone composition of the CP and investigate whether features such as the Crista Galli’s dimensions, or increased foramina area impact the bone composition of the CP.

Where medical facilities have limited access to 3D imaging software, future researchers may be able to generate a formula to apply the 3D imaging findings to 2D images to enhance the accuracy of these imaging methods. 2D imaging measurements could be compared with 3D imaging to explore the rate of the additional surface area measured with 3D imaging to generate this formula. The percentage of additional surface area could also be investigated with how it may correlate with other classifications such as the Keros (Keros 1962), Yenigun (Yenigun et al. 2016) or TMS (Abdullah et al. 2020) classifications.

Lastly, if a strong link could be determined between these CP structures and increased risk of fracture, individuals with a higher risk of facial fractures such as athletes in competitive sports (Povolotskiy et al. 2019) could receive an examination to determine whether their CP is at risk before engagement in the physical activity. This could result in at-risk individuals wearing appropriate headgear to prevent complications regarding the CP, and if assessed by insurance companies, policy premium could be more accurately moderated based on the risk of fractures of the individual.

## Data Availability

The data that support the findings of this study are not openly available due to reasons of sensitivity and are available from the corresponding author upon reasonable request.
